# Highly Efficient Air Sterilization via Low‐Temperature Interfacial Evaporation in Inductively Heated Superhydrophilic Ferromagnetic Filters

**DOI:** 10.1002/advs.202509118

**Published:** 2025-09-28

**Authors:** Arnau Fons, Cristina Vaca, Eloi Franco‐Trepat, Aritz Lafuente, José Luis Tajada, Albert Serrà, Jorge Diaz Pedroza, Sandra Franco, Rytis Boreika, Itziar Erkizia, Nuria Izquierdo‐Useros, Alberto López‐Ortega, Eneko Garaio, Maria Jose Esplandiu, Josep Nogues, Borja Sepúlveda

**Affiliations:** ^1^ Instituto de Microelectrónica de Barcelona (IMB‐CNM, CSIC) Campus UAB Barcelona 08193 Spain; ^2^ IrsiCaixa Germans Trias i Pujol Research Institute (IGTP) Universitat Autònoma de Barcelona (UAB) Barcelona 08916 Spain; ^3^ Catalan Institute of Nanoscience and Nanotechnology (ICN2), CSIC and BIST Campus UAB Barcelona 08193 Spain; ^4^ Grup d'Electrodeposició de Capes Primes i Nanoestructures (GE‐CPN) Departament de Ciència de Materials i Química Física Universitat de Barcelona Martí i Franquès, 1 Barcelona 08028 Spain; ^5^ Institut Germans Trias i Pujol Comparative Medicine and Bioimage Centre of Catalonia Barcelona 08916 Spain; ^6^ Instituto de Salud Carlos III CIBER Enfermedades Infecciosas (CIBERINFEC) Barcelona 28029 Spain; ^7^ Departamento de Ciencias Universidad Pública de Navarra Navarra 31006 Spain; ^8^ Institute for Advanced Materials and Mathematics (INAMAT²) Universidad Pública de Navarra Navarra 31006 Spain; ^9^ ICREA Pg. Lluís Companys 23 Barcelona 08010 Spain

**Keywords:** air sterilization, inductive heating, low‐temperature interfacial evaporation, pyrolytic self‐cleaning, superhydrophilic ferromagnetic filters, virus removal

## Abstract

The scientific evidence supporting airborne transmission of pathogens in closed spaces highlights the inefficiency of current air circulation and filtration technologies (e.g., HEPA filters, UV, ozone, ionization) in preventing the spread of airborne pathogens. This underscores the urgent need for new air disinfection devices. Here, the first air sterilization technology is presented based on low‐temperature interfacial evaporation. This novel approach integrates superhydrophilic micro/nano‐structured stainless‐steel filters and ultra‐efficient magnetic inductive heating to enable complete evaporation of water from the contaminated aerosols and the precipitation of all the organic and inorganic residues within the filter at temperatures in the range of 60–80 °C. The technology is validated through experiments with contaminated aerosols with different active viruses, including SARS‐CoV‐2 and respiratory syncytial virus (RSV), demonstrating the elimination of 99.6% or more of the nebulized viruses, at filter temperatures of 60–80 °C and airflow rates of 15 L min^−1^. The filters also support pyrolytic self‐cleaning and reuse, ensuring extended service time and minimal maintenance. This air sterilization technology represents a significant advancement over existing state‐of‐the‐art filtering technology, offering unmatched versatility, low energy consumption, and cost‐effective sterilization, without generating harmful radicals, dangerous high voltages, or high temperatures.

## Graphical abstract

The first low‐temperature interfacial evaporation air sterilization technology is presented. It is based on a superhydrophilic ferromagnetic micro/nano‐structured stainless‐steel filter, showing ultra‐efficient inductive heating. This technology has demonstrated over 99.6% of SARS‐CoV‐2 and RSV elimination at temperatures in the 60–80 °C range. The filters are reusable by self‐cleaning pyrolysis.

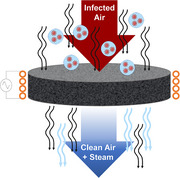

## 1 Introduction

Airborne pathogens, such as bacteria, viruses, fungi and parasites, pose a significant threat to public health, particularly in enclosed spaces such as healthcare facilities, public transportation, schools, and offices. The ability to effectively disinfect the air is crucial in preventing the spread of infectious diseases. Several air disinfection methods have been developed,^[^ ^1^ ^]^ including passive filtration, ultraviolet radiation, ozone, plasma^[^ ^2^ ^]^ and thermal deactivation.

Current passive air filtering systems in sterile spaces, passenger cabins, and many air conditioning systems, are based on High Efficiency Particle Arresting (HEPA) filters, which mechanically trap the pollutants through mechanisms such as interception, inertial impact, diffusion, and gravity, but they do not sterilize the air.^[^ ^3^, ^4^ ^]^ Moreover, HEPA filters do not guarantee complete filtration of small viral particles, and filters can become a biological hazard due to the accumulation of active bioelements, acting as a secondary source of contamination.^[^ ^5^, ^6^, ^7^ ^]^ Thus, HEPA filters must be replaced and disposed periodically. These filters also degrade over time, compromising their efficiency, and they cannot be decontaminated and reused, contributing to environmental waste.^[^ ^8^ ^]^ Finally, HEPA filters cannot withstand high temperatures and high air pressures.^[^ ^6^ ^]^

Another widespread active method for inactivating pathogens in the air is ultraviolet germicidal irradiation (UVGI).^[^ ^9^ ^]^ UVGI damages DNA/RNA of pathogens, decreasing their replication rate and leading to their extinction.^[^ ^10^ ^]^ However, the application of UVGI has raised concerns related to potential injury to humans since any leakage of UV radiation can pose serious health risks (specifically to eyes and skin), in addition to generating harmful ozone.^[^ ^11^ ^]^ Mercury‐based ultraviolet lamps, the most common UVGI devices, pose additional safety and environmental risks due to the potential release of hazardous mercury in case of lamp breakage. Light‐emitting diodes (LEDs) are an alternative approach to replace conventional mercury‐containing ultraviolet lamps. However, at present, LED‐based ultraviolet air disinfection systems are limited by their low light power. Consequently, their practical deployment often relies on hybrid solutions combining LED irradiation with complementary technologies, such as HEPA filtration and/or additional UVGI sources, to achieve the inactivation levels required in real‐world heating‐ventilation‐air conditioning (HVAC) applications. Moreover, UVGI effectiveness is strongly influenced by the intensity of the UV light and the duration of exposure, as some pathogens are inactivated only by high UV doses or long exposure times.^[^ ^4^, ^12^ ^]^ Additionally, UVGI does not remove biological by‐products like endotoxins, which can still pose health risks.^[^ ^13^ ^]^

Other recent developments in air filtration technology include self‐cleaning particulate matter filters employing sunlight‐driven photocatalysis or microwave‐responsive MXene coatings for viral inactivation.^[^ ^14^, ^15^ ^]^ These systems utilize advanced materials, but are highly dependent on environmental conditions, such as sunlight availability or continuous microwave exposure, restricting their scalability and continuous efficacy. Nanofiber‐based filters, despite their high filtration efficiency, suffer from rapid fouling, electrostatic charge loss, and efficiency reductions at varying airflow velocities.^[^ ^16^ ^]^ Additionally, antiviral textiles utilizing metal, polymer, or carbon nanomaterials are limited due to safety concerns, environmental risks, high production costs and limited time of action on the pathogen targets.^[^ ^17^ ^]^ Other innovative surface‐based strategies, such as nanostructured surfaces with virucidal topographies (nanopillars), show antiviral effects through mechanical piercing. Nevertheless, their effectiveness requires prolonged virus‐surface contact (>6 h), rendering them impractical for rapidly circulating air.^[^ ^18^ ^]^

In contrast, thermal air disinfection has emerged as a promising alternative due to its simplicity, efficiency, and broad potential application.^[^ ^19^ ^]^ Thermal air disinfection involves heating air to a temperature high enough to inactivate microorganisms. This method is based on the principle that most pathogens are sensitive to heat and can be killed or inactivated at elevated temperatures. The effectiveness of thermal air disinfection depends on factors such as temperature, exposure time, and the type of microorganisms.^[^ ^20^ ^]^ Currently, various thermal treatment methods for disinfecting pathogens have been demonstrated. For example, electrically conductive carbon nanotube filters were developed to prevent problems of bioaccumulation, by generating resistive heating to deactivate the trapped biohazards.^[^ ^21^, ^22^ ^]^ However, their complexity, high‐cost, limited scalability, and concerns regarding potential particle release, may pose health risks a prevent their practical application.^[^ ^13^, ^22^ ^]^ Alternatively, copper nanowire air filters effectively captured particulate matter by mechanical and electrostatic filtration mechanisms, enabling subsequent elimination of trapped bacteria by Joule heating at 100 °C during 10 min.^[^ ^23^, ^24^ ^]^ However, this system has not been demonstrated with smaller pathogens like viruses, and the low stability of the Cu nanowires can compromise the long‐term applicability. Additionally, Yu et al. developed resistively heated nickel foams for thermal air disinfection.^[^ ^25^ ^]^ They demonstrated 99.8% of SARS‐CoV‐2 deactivation in one pass of aerosol at a filter temperature of 200 °C under an airflow of 10 L min^−1^ using a Ni foam with a thickness of 1.6 cm. They also achieved an inactivation efficiency of 99.9% of the airborne bacterium Bacillus anthracis. However, the large size, high cost, and potential environmental impact of Ni foams can limit their commercial application. Therefore, there is a need to find low‐cost, energy‐efficient, scalable, and reusable thermal air disinfection systems.

On the other hand, interfacial water evaporation has gained significant attention in recent years as a promising technique for water remediation. Its energy efficiency and cost‐effectiveness make it a compelling alternative to traditional methods.^[^ ^26^ ^]^ By leveraging the enhanced heat transfer at the water‐material interface, the interfacial evaporation can efficiently remove contaminants from water at temperatures well below the boiling point, resulting in clean, distilled water.

Here, we show a novel cost and energy‐efficient concept for air disinfection based on low‐temperature interfacial evaporation using inductively heated superhydrophilic ferromagnetic stainless‐steel filters.^[^ ^27^, ^28^ ^]^ We first describe the thermal modification process of the stainless‐steel filters to simultaneously induce superhydrophilic and ferromagnetic behavior. Next, we analyze the capacity of the inductively heated filters to evaporate aerosols containing salts, proteins, liposomes mimicking virus, organic pollutants or polymer nanoparticles, yielding complete precipitation of all the organic and inorganic content inside the filter at temperatures below the boiling point of water. Exploiting this mechanism, we demonstrate the capacity to completely sterilize the airflow contaminated with large concentrations of SARS‐CoV‐2 pseudovirus, SARS‐CoV‐2 omicron virus, or respiratory syncytial virus (RSV) aerosols, keeping the filters at temperatures in the 60 °C to 80 °C range under an airflow of 15 L min^−1^. Finally, we show that a pyrolytic cleaning process can completely regenerate the filter's surface. This method enables the filters to be reused for a minimum of 10 consecutive cycles.

## 2 Results and Discussion

### 2.1 Air Sterilization Working Principle

**Figure 1:**
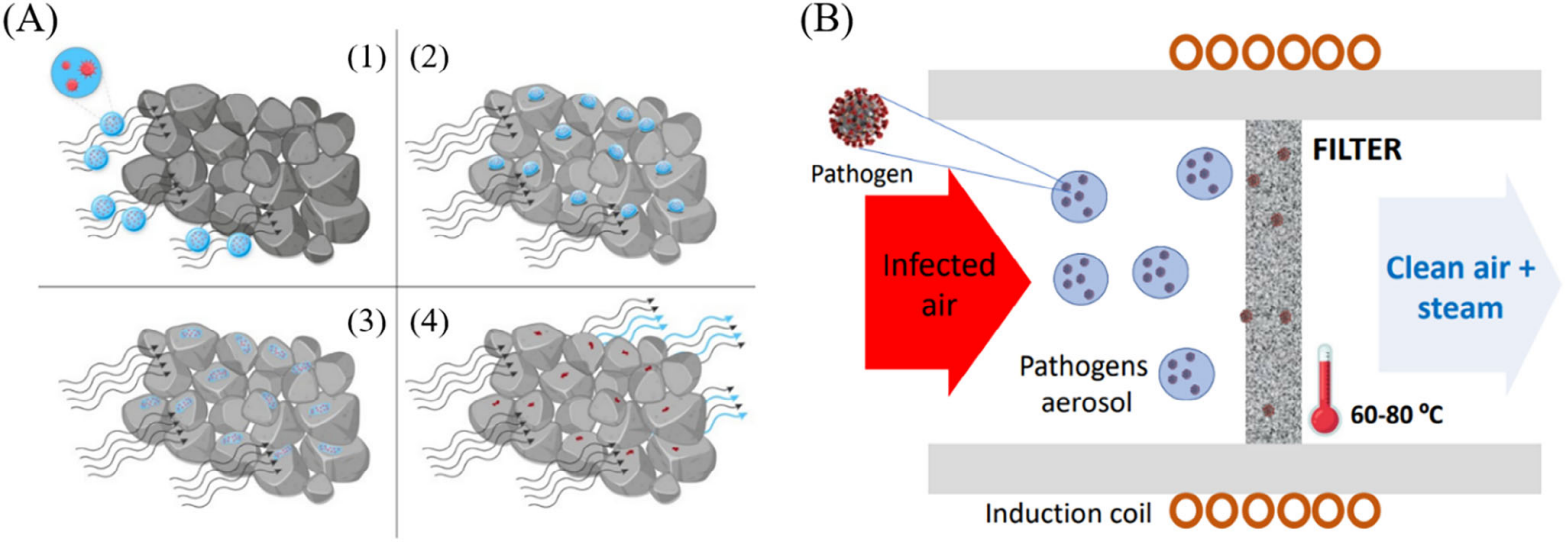
Air disinfection working principle. A) Schematic of the interfacial water evaporation process: (1,2) the aerosol microdroplets collide with the mildly heated superhydrophilic stainless‐steel microparticles of the filter, (3) the aerosol microdroplets rapidly spread over the metal surface, thus increasing the surface contact area with both metal and air, and (4) the enhanced heat transfer and air/water interface area induce a fast evaporation of the water molecules and the precipitation of the organic and inorganic content. B) Schematic of the mildly heated SFSSM filters by magnetic induction inside an air pipe, showing the incident infected air and, at the output, the clean air plus steam.

The working principle of the air sterilization device is based on the low‐temperature interfacial liquid evaporation, as follows (**Figure** **1**A,B). The contaminated airflow with aerosol micro‐droplets containing the pathogens goes through the superhydrophilic and ferromagnetic stainless steel microporous (SFSSM) filter that is mildly heated by magnetic induction to a temperature between 60 °C and 80 °C. During this process, the aerosol micro‐droplets collide with the superhydrophilic filter (Figure 1A, panels 1‐2), forcing the liquid to spread over its nanostructured surface (Figure 1A, panel 3). Such liquid spreading enables the rapid and complete evaporation of the water molecules at temperatures well below boiling, thereby precipitating all their inorganic and organic content (salts, pathogens, proteins…) on the filter surface (Figure 1A, panel 4). As a result, at the filter output, only clean sterilized air and water vapor are obtained (Figure 1B). Due to the low filter temperature and short residence time of the air inside the filter (ca. 2–3 ms), the air temperature increase at the output is minimal.

### 2.2 Structure and Properties of the Treated SFSSM Filters

**Figure 2:**
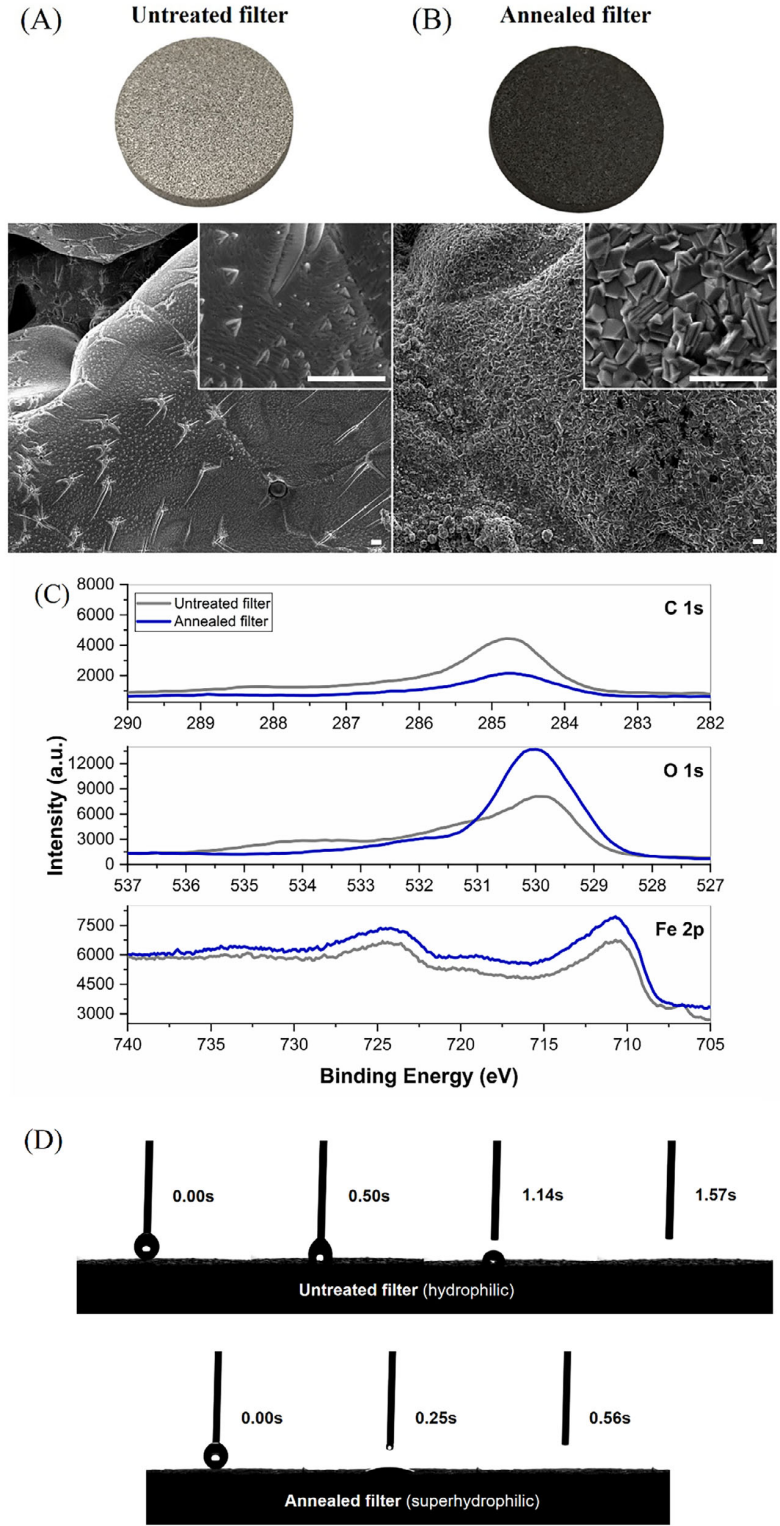
Structural characterization of the untreated and annealed filters and wettability analysis. A,B) Photographs and SEM images of the annealed and untreated filters (Scale bars 2 µm). C) XPS of the annealed and untreated filters showing the C1s, O1s, and Fe2p peaks. D) Analysis of the hydrophilicity of the annealed and untreated filters by quantification of the droplet absorption time.

The core of the air sterilization device are stainless (AISI 316L) steel microporous filters (Amespore®) with a diameter of 20 mm, thickness of 3 mm, and an average pore size of 10 µm (**Figure** **2**A,B). To simultaneously maximize their hydrophilicity and the inductive heating efficiency, the filters were subjected to a thermal treatment in atmospheric conditions at 950 °C for 1 h. During this process, the filter surface became nanostructured (Figure 2A,B). According to Brunauer‐Emmett‐Teller (BET) analysis, the specific surface area significantly increased after the annealing from 0.236 to 0.495 m^2^ g^−1^ (ca. two‐fold increase). The X‐ray photoelectron spectroscopy (XPS) analysis (Figure 2C) showed that the annealed filter surface presented a higher concentration of iron and oxygen compared to the untreated sample, which is attributed to the continuous growth of iron oxides during the annealing. In addition, the carbon C1s spectrum decreased due to its oxidation to carbon dioxide. The high‐resolution spectra of O1s and Fe2p of the untreated filter exhibited a small metallic signal Fe 2p_3/__2_ ≈707 eV, oxide components at 710–711 eV for the 2p_3_/_2_, as well as the 2p_1_/_2_ oxides signal at 724.5 eV. In contrast, metallic iron component of the annealed filter was completely absent, showing only the Fe_2_O_3_ signatures at 710–711 and 724.5 eV, corresponding to the core levels of 2p_3/__2_ and 2p_1/__2_, respectively.

These morphological and chemical surface changes also modified the filters hydrophilicity. The contact angle could not be determined because the water droplets were completely absorbed by the filter. However, the droplet absorption time was drastically reduced four‐fold in the annealed filters, from ≈1.1 to 0.25 s (Figure 2D), thereby demonstrating the change from hydrophilic to superhydrophilic behavior. This effect was due to the inherent high hydrophilicity of the iron oxide surfaces, and the two‐fold increase of the surface area induced by the annealing.

**Figure 3:**
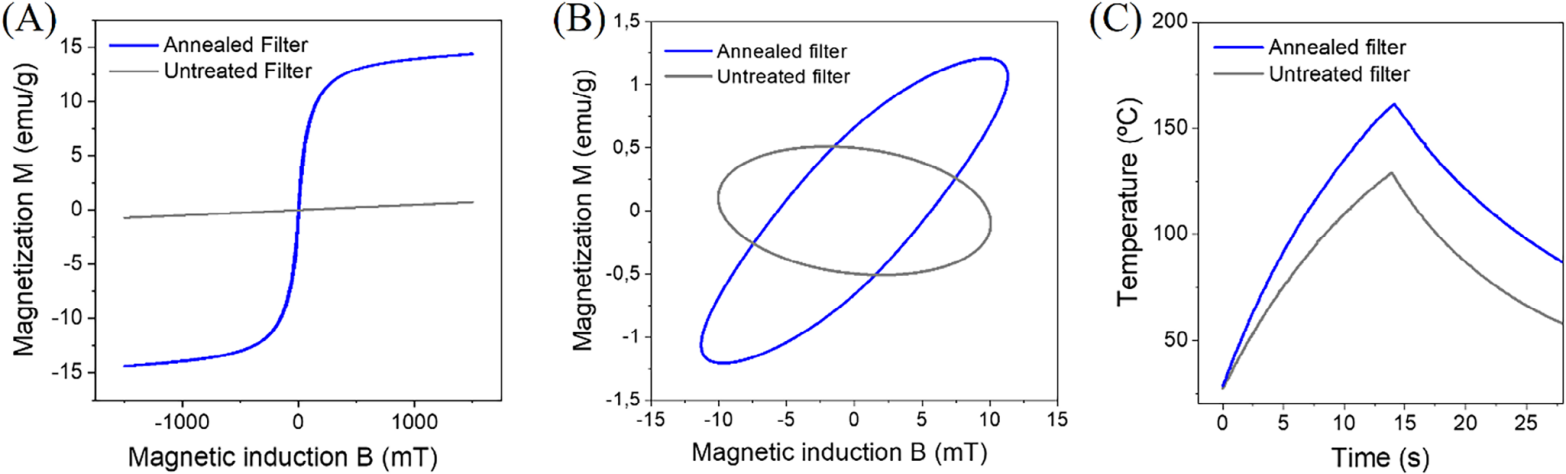
Magnetic properties of the SFSSM filters. A) DC magnetic properties of the untreated and annealed filters, showing the paramagnetic to ferromagnetic transformation. B) AC magnetization hysteresis curves at 78 kHz of the untreated and annealed filters. C) Demonstration of the enhanced induction heating efficiency in the treated filters under alternating magnetic field (40 mT and 92.5 kHz) and airflow of 15 L min^−1^, compared to the untreated filters.

The annealing process also significantly modified the magnetic properties of the filters. As can be observed in **Figure** **3**, the magnetization curve of the filters changed from paramagnetic to ferromagnetic behavior with low coercivity (ca. 5 mT) and saturation magnetization of ca. 15 emu g^−1^. The modification of the magnetic response was also reflected in the AC magnetometry measurements (Figure 3B). At a magnetic field frequency of 78 kHz, the untreated filters exhibited the expected ellipsoidal magnetization loop with negative slope inclination, which is typical of the induced Eddy currents in the filter. In contrast, the annealed filters showed an ellipsoidal magnetization loop with a positive slope and higher ellipsoid area, as a result of the ferromagnetic transformation induced by the thermal process. A similar effect was observed at a magnetic frequency of 130 kHz. This AC magnetization change is highly relevant to increase the induction heating efficiency. This effect is illustrated in Figure 3C, which shows a 35% higher temperature increase in the annealed filters compared to the untreated ones, when exposed to an alternating magnetic field of 40 mT at 92.5 kHz, under an airflow of 15 L min^−1^ passing through the filters. Interestingly, when the airflow was raised to 20 L min^−1^, the magnetically induced temperature increase in the treated filters was even higher, ca. 45% higher than the untreated filters (Figure S1, Supporting Information). Due to the limitations of the air pump system, the subsequent assays were carried out at 15 L min^−1^.

### 2.3 Air Sterilization Prototype with Integrated Nebulization and Liquid Collection Chambers

**Figure 4:**
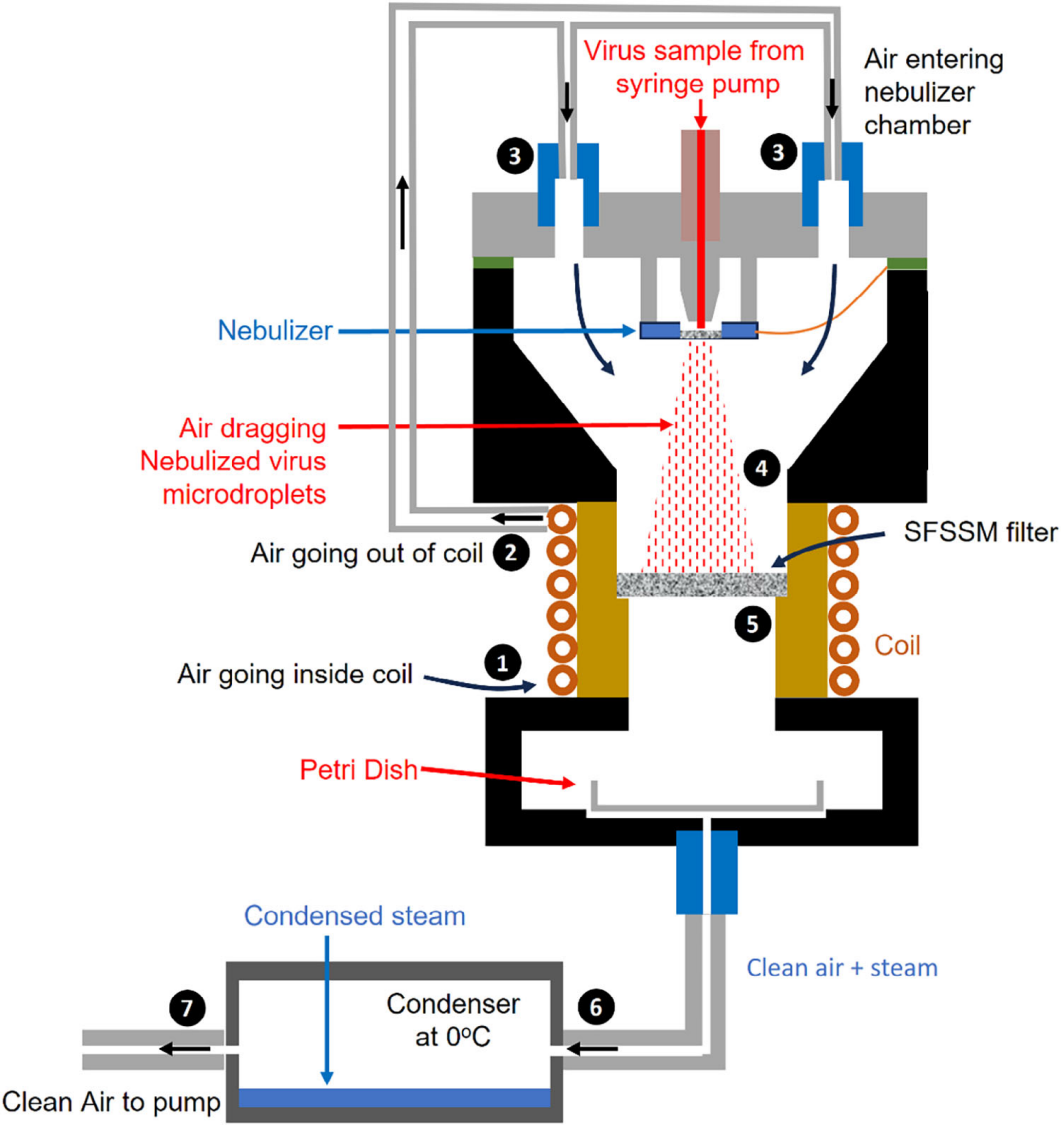
Schematic of the air sterilization prototype for the analysis of the air disinfection efficiency and description of the operation sequence. The ambient air sucked by the vacuum pump enters the hollow coil (1) to cool it down. The air at the coil exit (2) is introduced in the nebulization chamber (3) by two equidistant ports to drag the aerosol microdroplets. The contaminated air with the aerosol (4) goes through the SFSSM filter (5), where the aerosol microdroplets are evaporated by the low temperature interfacial evaporation process, thus precipitating all the organic and inorganic contents in the filter (5). The clean air and the produced steam enter the condenser chamber to condense part of the steam (6). The clean air finally exits through the vacuum pump (7).

To analyze the air sterilization efficiency of the treated SFSSM filters, we developed an induction heating prototype working at 40 mT and 92.5 kHz, integrating a custom‐made piezoelectric nebulizer chamber (**Figure** **4**). The inductive heater was composed of a hollow coil (6 turns), which was driven by a resonant LC circuit. The maximum AC power consumption of the system was 150 W. To monitor and control the filter temperature, the nebulizer chamber incorporated an infrared transparent ZnSe window, and an infrared thermometer pointing to the filter surface. To control the filter temperature, the inductor was programmed with ON/OFF steps of variable duration to stabilize the filter at different temperature ranges. The treated filter was located inside a temperature‐resistant PEEK holder. This holder was hermetically connected to the nebulizer and fluid collection chambers.

**Figure 5:**
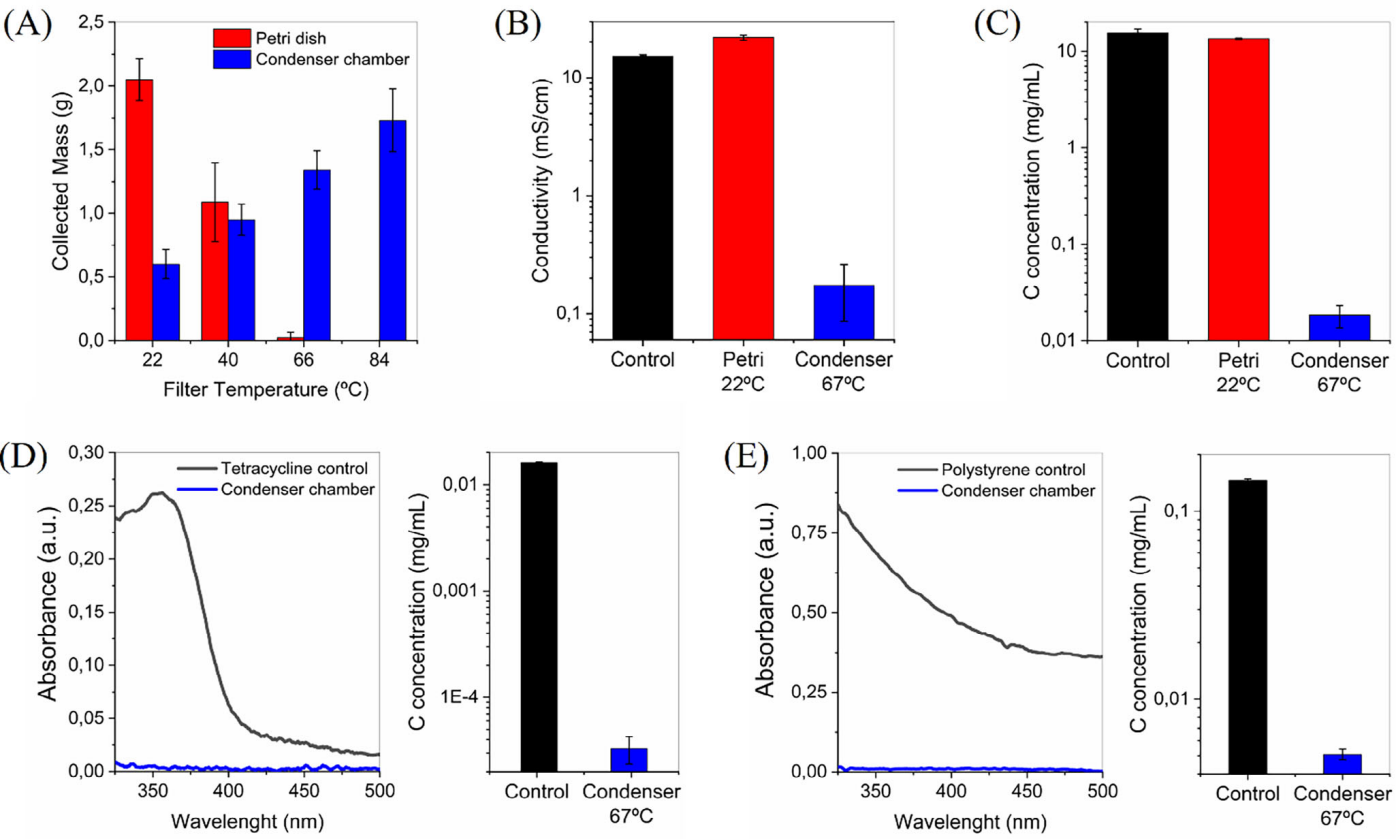
Analysis of the collected fluids in the Petri dish and condenser chamber under different filter temperatures, airflow rates, and nebulized fluid compositions. A) Mass of sample collected in the Petri dish and condenser chamber as a function of the filter temperature after nebulizing 3 mL of cell medium with serum. B) Conductivity of the samples collected in the Petri dish and condenser chamber at filter temperatures of 22 °C and 67 °C, when PBS buffer was nebulized. C) Total organic content (TOC) in the samples collected in the Petri dish and condenser chamber at filter temperatures of 22 °C and 67 °C, when cell medium supplemented with serum and liposomes was nebulized. D) Absorbance and TOC values of a solution containing 16 ppm of tetracycline before and after the filtering process. E) Absorbance and TOC values of a solution containing 100 µg mL^−1^ of carboxylate polystyrene nanoparticles before and after the filtering process. All the experiments were conducted by nebulizing the samples at 0.25 mL min^−1^ under an airflow of 15 L min^−1^.

**Figure 6:**
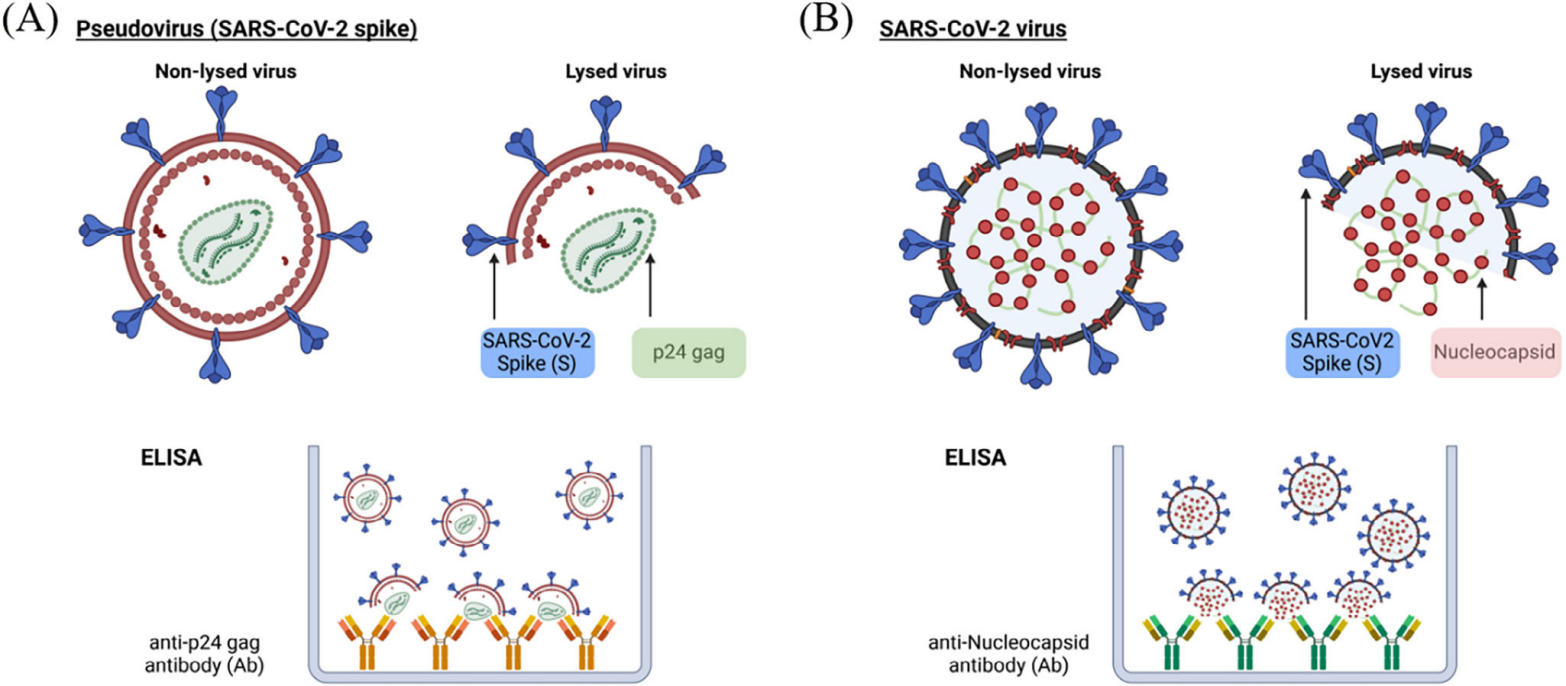
Schematics of the analyzed virus and their detection process. Illustrations of A) the SARS‐CoV‐2 pseudovirus and B) the SARS‐CoV‐2 virus before and after lysing for detection via ELISA assay.

The nebulizer chamber integrated a piezoelectric nebulizer with a metal mesh featuring an array of holes with 2 µm diameter. This system was designed for the nebulization of small pathogens, such as viruses, and prevents the analysis of larger pathogens (e.g., bacteria, or fungi). The nebulizer was coupled to a syringe pump filled with the pathogen sample to control the injection flow rate. The airflow speed was controlled with a vacuum pump and a manual flow meter. The nebulizer chamber had two air inlets, which were coupled to the hollow coil, thereby acting as an intercooler. In this configuration, the ambient air from the laboratory passed first through the coil (Figure 4‐1) to cool it down, and to slightly increase the air temperature before entering the nebulizer chamber (Figure 4‐2,3). The air entering the nebulizer chamber dragged the aerosol droplets toward the filter (Figure 4‐4). The contaminated air with the aerosol microdroplets passed through the treated SFSSM filters (Figure 4‐5), and the liquid that was not evaporated during this process was collected in a plastic Petri dish (diameter 3.5 cm) located in the collection chamber below the filter. Finally, to analyze the composition of the evaporated liquid, the air passed through a stainless‐steel chamber maintained at 0 °C inside a water/ice bath to condense a large fraction (>50%) of the evaporated fluid (Figure 4‐6).

Note that this complex setup was designed merely to test the pathogen elimination efficiency of the system. In real air purification applications, the configuration would be considerably simpler (Figure 1), as the nebulizer and condenser chambers are not necessary. In fact, this type of device would be easily adaptable to any type of air pipe for a wide range of applications. Additionally, in contrast to most of the existing air purification schemes, the inductive‐heated SFSSM filters withstand much higher air pressures and temperatures.

### 2.4 Optimization of the Filter Temperature and Analysis of the Collected Liquids

To determine the optimal thermal actuation conditions for efficient air disinfection, we first evaluated the evaporation efficiency of nebulized microdroplets composed of various fluids mimicking pathogenic aerosols. We analyzed the composition of liquids collected in both the Petri dish and the condenser chamber as a function of filter temperature, while maintaining a constant airflow of 15 L min^−1^ (**Figure** **5**). The contaminated airflow used in these experiments was composed of the nebulized liquid microdroplets combined with atmospheric air from the laboratory. Therefore, the airflow could have contained, in addition to the nebulized microdroplets, the typical airborne pollutants, pathogens, and pollen found in the Universitat Autònoma de Barcelona campus region. We first quantified the mass of sample collected in the Petri dish and the condenser chamber after nebulizing 3 mL (ca. 3 g) of cell medium with 10% of serum at an injection rate of 0.25 mL min^−1^. As can be seen in Figure 5A, the mass collected in the Petri dish rapidly decreased as the filter temperature increased due to the efficient evaporation of the aerosol microdroplets, being already nearly zero at a filter temperature of 60 °C. At the same time, the quantity of water collected in the condenser increased due to the efficient interfacial water evaporation. For temperatures between 60 °C and 80 °C, the amount of collected water was ca. 60% of the nebulized mass. However, the collection efficiency could be readily enhanced by employing a serpentine condenser to increase heat exchange with the cold bath, thereby achieving nearly complete water recovery. Therefore, at temperatures over 60 °C all the salts and organic compounds of the cell medium and serum precipitated in the filter, and, consequently, the collected liquid in the condenser should be clean distilled water.

To illustrate this, we analyzed the conductivity of the recovered liquid in the Petri dish and condenser chamber. To avoid the interference of the proteins in the conductivity measurements, the liquid in this case was phosphate‐buffered saline (PBS 1X), nebulized at 0.25 mL min^−1^. As Figure 5B shows, when the filter temperature was 67 °C, the conductivity of the liquid in the condenser chamber was ca. two orders of magnitude lower than the nebulized liquid. In contrast, the conductivity of the liquid collected in the Petri dish at room temperature was slightly higher than the control, which reflects a partial evaporation of the aerosol microdroplets even at room temperature.

To demonstrate that also the organic compounds precipitated in the filter, we analyzed the total organic content (TOC) in the liquids collected in the Petri dish and condenser chamber. In this case, to mimic the viral particles, we nebulized a solution containing cell medium supplemented with serum and liposomes with an average diameter of 100 nm, i.e., similar to typical virus sizes, at a concentration of 125 µM. As Figure 5C shows, the TOC in the condensed liquid was nearly 3 orders of magnitude lower than the control and the Petri dish at room temperature, despite the large concentration of organic compounds in the nebulized sample (ca. 15 mg mL^−1^). Therefore, the collected liquid in the condenser chamber was clean distilled water, and the actuation conditions yielding complete evaporation of the nebulized droplets should induce a complete sterilization of the air flowing through the filter.

To demonstrate that the low temperature interfacial evaporation process can also eliminate other non‐volatile contaminants, the following contaminated samples were nebulized containing: i) the antibiotic tetracycline, which is an important environmental pollutant, and ii) negatively changed polystyrene nanoparticles (diameter 100 nm), mimicking microplastics. All the experiments were carried out with a filter temperature between 60 °C and 80 °C, and airflow rate of 15 L min^−1^. At these operation conditions, the liquid collected in the Petri dish was always zero. To analyze the tetracycline removal, the variation of its UV absorption band with a peak at 360 nm was monitored. As can be observed in Figure 5D, the absorbance peak completely disappeared in the condensed liquid. To demonstrate that the drastic absorbance decrease was not due to the partial degradation of the pollutant, the TOC of the condensed samples was also analyzed. The TOC removal was in the same range (average 99.79%) as the previously analyzed samples with cell medium, serum, and liposomes, thereby agreeing with the collection of clean distilled water. In contrast, the elimination of negative polystyrene nanoparticles is more challenging due to their high hydrophobicity and the electrostatic repulsion with the filter surface. Consequently, the interaction of the particles with the filter is weak and the precipitated particles can be dragged by the airflow. Despite these difficulties, a remarkable TOC removal of 96.56% was achieved, as can be seen in Figure 5E, which was corroborated by a high absorbance reduction.

All these experiments were carried out in a laboratory without humidity control. The humidity at the Universitat Autònoma de Barcelona campus region typically varies between 60% and near 90% throughout the year. However, we have not observed any noticeable change in the interfacial evaporation efficiencies with respect to the relative humidity variations. Actually, the interfacial evaporation process should be even more efficient in regions with lower relative humidity, thereby probably enabling a reduction of the filter temperature to achieve the full evaporation of the aerosol microdroplets.

The energy consumption of the air sterilization system is also a relevant metric. A cycle of 1‐second inductive heating pulses at 150 W, followed by 4 s with the power off, was used to reach the required filter temperature of ≈65 °C. Therefore, this approach enabled complete aerosol evaporation and precipitation within the filter, with an average power consumption of only 30 W.

These capabilities of the SFSSM filters, combined with a more efficient water condensation system, could find interesting applications for low volume water purification. However, it is worth noting that the interfacial evaporation process would not eliminate pollutants with higher volatility than water, such as benzene, toluene, and so on.^[^ ^26^ ^]^

### 2.5 Analysis of Air Disinfection from SARS‐CoV‐2 Pseudoviruses and Infectious Viruses

To demonstrate the complete air disinfection through low‐temperature interfacial evaporation, we assessed the air sterilization ability of the SFSSM filters after nebulization of three distinct viral agents: i) SARS‐CoV‐2 pseudovirus (constructed by pseudotyping HIV‐1 particles with SARS‐CoV‐2 spike), ii) replication competent SARS‐CoV‐2 virus (Omicron XBB.1.5) or iii) respiratory syncytial virus (RSV) tagged with green florescence protein (GFP). In these assays we kept filter temperatures between 60 °C and 80 °C, ensuring complete liquid evaporation under a nebulization speed of 0.25 mL min^−1^ and an airflow rate of 15 L min^−1^. As in the previous characterization experiments, before starting the nebulization, the filters were heated for 7 s to reach a temperature within the 60 °C and 80 °C range, and cycles of heating pulses of 1 s, followed by 4 s without heating were used to keep the temperature within that range (average consumed power of 30 W). These experiments were carried out at the BSL3 biosafety laboratory of the Comparative Medicine and Bioimage Centre of Catalonia (CMCIB). In this case, as the pumped air from the BSL3 lab was filtered to avoid any external contamination, the airflow only contained, as contaminants, the nebulized microdroplets with the virus.

We quantified the presence of the pseudovirus or virus in liquid samples collected from the Petri dish or condenser chamber by ELISA. In the case of SARS‐CoV‐2 pseudovirus, we determined the concentration of the HIV viral protein p24 gag. For the SARS‐CoV‐2 virus, we measured the concentration of viral protein nucleocapsid, and the RSV was detected by measuring the GFP protein. In intact viruses that are infectious, these viral proteins are located internally within the membranes of these enveloped viruses. Therefore, the quantification was performed in the presence and absence of a lysis buffer that disaggregates the viral membranes to detect and quantify the internal proteins (**Figure** **6**).

First, to demonstrate that the nebulizer and the filter do not affect the viral integrity, 2 mL of the SARS‐CoV‐2 pseudovirus was nebulized at 0.25 mL min^−1^ under an airflow of 15 L min^−1^ with the filter at room temperature. The concentration of the p24 gag protein from the liquid samples collected in the Petri dish was analyzed. As Supporting Table S1 (Supporting Information) shows, the relative concentration of the p24 gag proteins before and after lysing, compared to the control, was nearly equal, thereby ensuring the integrity of the virus during the nebulizing process. The slightly higher concentration observed in the collected liquid in the Petri dish was due to the partial evaporation of the aerosol droplets during their travel through the filter.

**Figure 7:**
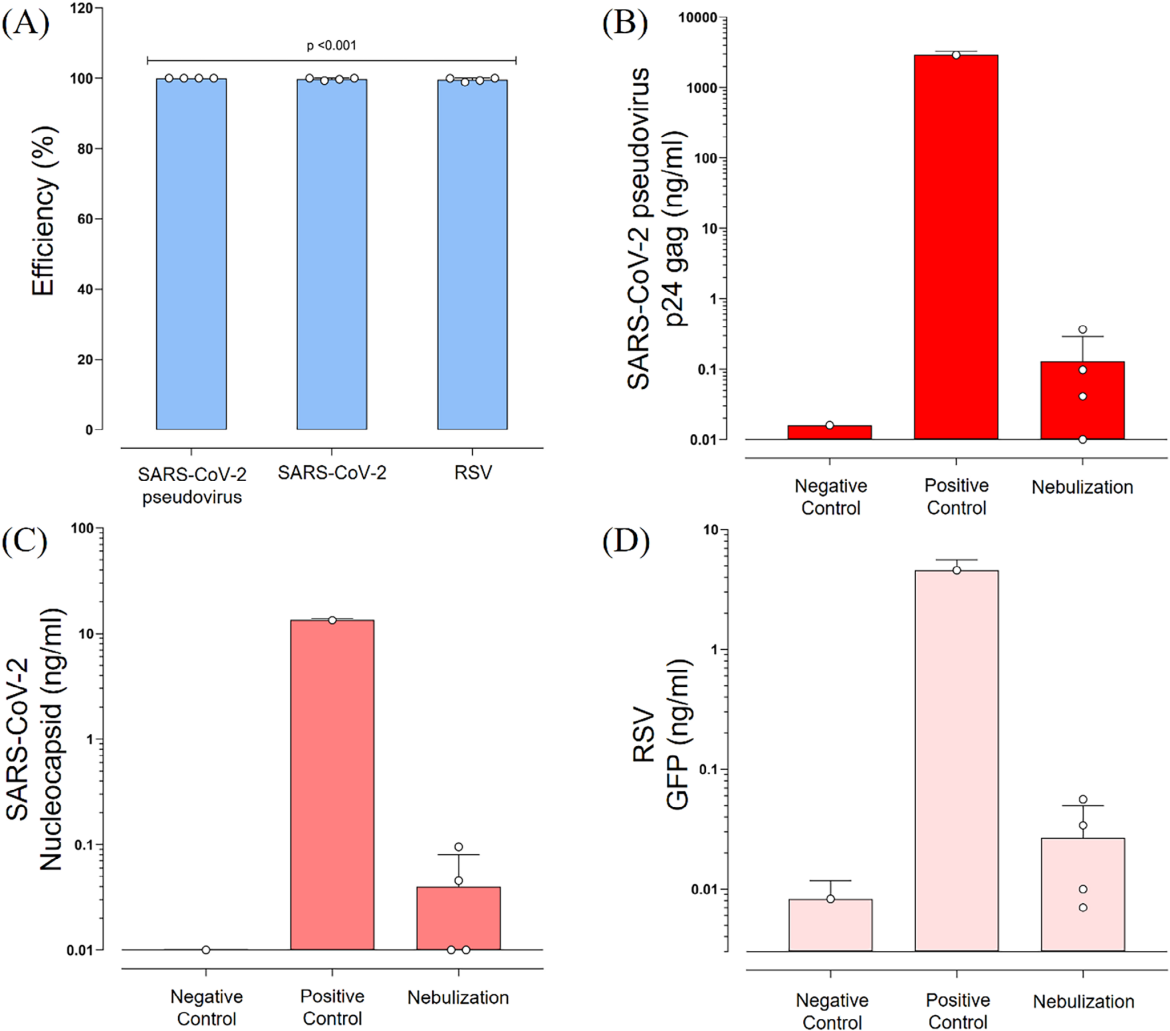
Results of the air disinfection from contaminated air with active virus when filters were heated at temperatures between 60 °C and 80 °C, under an airflow of 15 L min^−1^, and nebulized aerosols at 0.25 mL min^−1^. A) Percentage of viral elimination efficiency of the heated SFSSM filters compared to non‐nebulized controls. B) SARS‐CoV‐2 pseudovirus quantification by ELISA detection of p24 gag protein. C) SARS‐CoV‐2 XBB.1.5 Omicron variant quantification by ELISA detection of nucleocapsid. D) RSV quantification by ELISA detection of GFP protein. Statistical differences were calculated with a one‐sample t‐test. Bar graphs show the Mean ± SD from experimental replicas (*n* = 1 to 4), and dots represent the mean from technical replicas (*n* = 2 to 4).

Next, we nebulized 2 mL samples of SARS‐CoV‐2 pseudovirus under the same airflow and nebulization conditions, but the filter temperature was set between 60 °C and 80 °C. The p24 gag protein concentration was analyzed and, as expected at this filter temperature, the collected mass in the Petri dish was zero due to the complete evaporation of the aerosol droplets. In contrast, ca. 700 µL of liquid was collected in the condenser chamber. Remarkably, the concentrations of p24 gag protein in the recovered condensed liquid after the lysis buffer were below the detection limit of the ELISA (ca. 0.1 ng mL^−1^) (Table S2, Supporting Information). On average, the p24 gag viral protein elimination had an efficiency of 99.9998% (**Figure** **7**A). As can be observed in Figure 7B and Table S2 (Supporting Information) the protein concentration was nearly four orders of magnitude lower than the control and, in several measurements, the detection was zero.

To further confirm the air disinfection ability of the SFSSM filters, 2 mL samples containing SARS‐CoV‐2 XBB.1.5 Omicron virus were nebulized under the same airflow and filter temperature conditions. The concentration of viral nucleocapsid protein was analyzed by ELISA from the liquid collected in the condenser, as the mass collected in the Petri dish was also zero. On average, the viral nucleocapsid protein elimination had an efficiency of 99.8% (Figure 7A). As can be seen in Figure 7C and Table S3 (Supporting Information), in most of the nebulization experiments, the nucleocapsid was completely undetected (6 out of 8). Only in two replicas the nucleocapsid presence was found in values below the detection limit of the assay. Similar results were obtained using RSV, as a different type of enveloped virus, under the same airflow and filter temperature conditions. In this case, the average removal efficiency was 99.6% (Figure 7A,D; Table S4, Supporting Information), with 5 out of 8 measurements being zero. These results corroborate the efficiency of the low‐temperature interfacial evaporation concept to trap the viral particles in the aerosols, even at large concentrations.

### 2.6 Filters Pyrolytic Cleaning, Lifetime, Cost, and Scaling Up Analysis

Another key feature of the SFSSM filters is their self‐cleaning capability enabling safe reuse. The self‐cleaning process relies on the robustness, thermal resistance, and efficient inductive heating of the SFSSM filters. Due to the interfacial water evaporation process, the trapped pathogens in the filters should be dehydrated and, therefore, deactivated on the warm filter surface. However, before filter removal for pyrolytic cleaning, a short thermal treatment at higher temperature (e.g., 150 °C) without airflow would be advisable to ensure the complete denaturization of the trapped pathogens.

**Figure 8:**
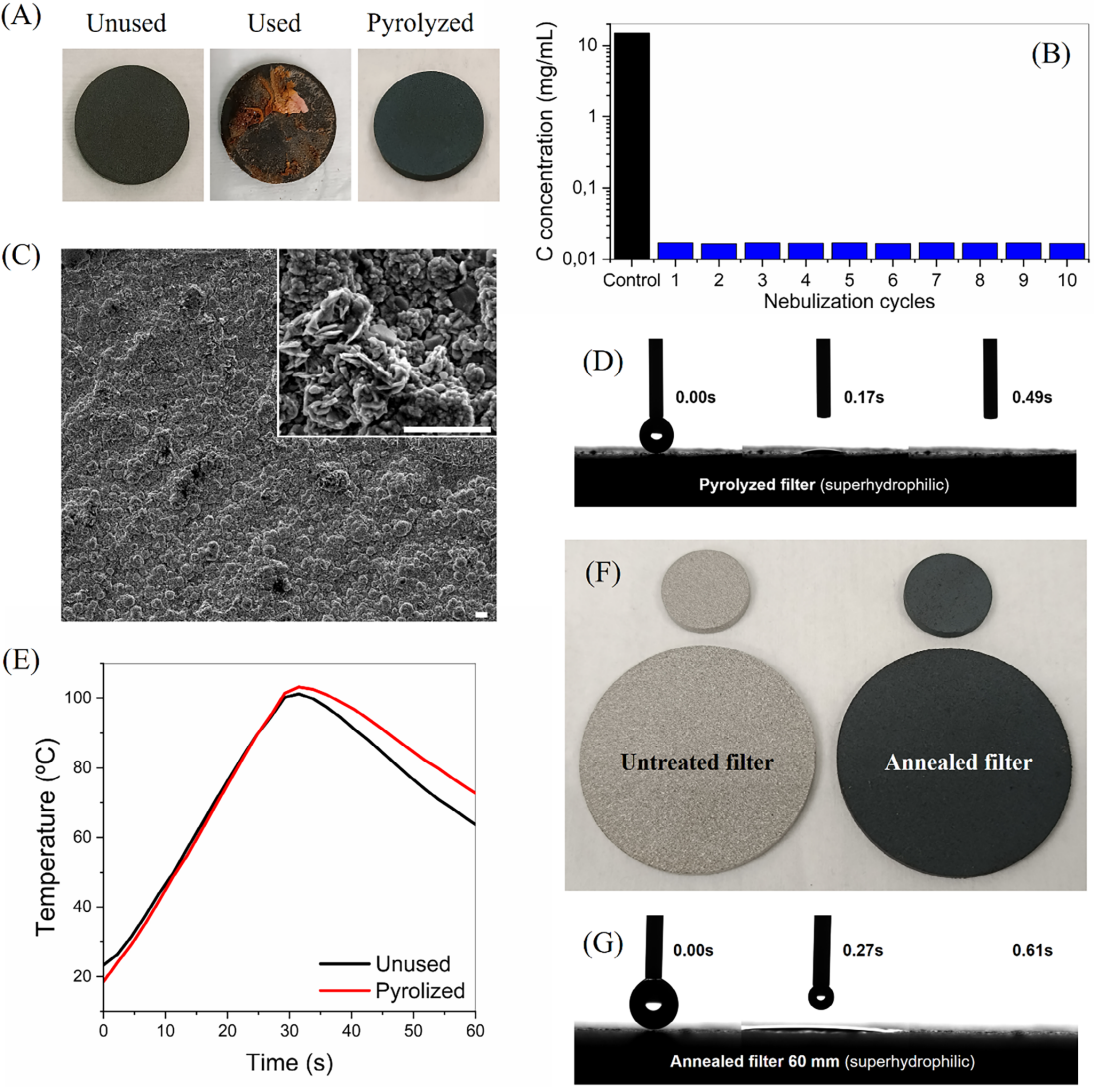
Filters pyrolytic cleaning and reuse. A) Photographs of the unused, used, and pyrolytically cleaned filters. B) TOC values of the liquid collected in the condenser chamber after pyrolytic filter cleaning for 10 consecutive times. C) SEM image of the pyrolytically cleaned filter after 10 pyrolytic cleaning processes (Scale bars 2 µm), showing an increase of surface nanostructuration compared to the unused filters. D) Wettability of the pyrolytically cleaned filters showing enhanced superhydrophilic behavior. E) Comparison of the inductive heating curves for the unused and pyrolytically cleaned filters. F) Pictures of the 6 cm diameter untreated and annealed filters compared to the 20 mm filters. G) Wettability of 6 mm diameter annealed filter, showing the superhydrophilic behavior.

To show the filters reusability, 6 mL samples of cell medium supplemented with serum and liposomes were nebulized at 0.25 mL min^−1^ under an airflow of 15 L min^−1^, keeping the filter at a temperature of between 60 °C and 80 °C to ensure the full liquid evaporation. As can be observed in **Figure** **8**A (Used), due to the large amount of nebulized medium, the deposited organic and inorganic residues were observed by the naked eye. To achieve a full recovery, the filters were subjected to a pyrolytic process at 900 °C for just 1 min by induction heating. This process yielded a completely clean surface, even from the precipitated salts. However, when the heating process was not completely homogeneous, it generated slight mechanical deformation in the filters, which could prevent further cleaning processes.

To minimize the cleaning temperature and the risk of mechanical deformation of the filters during the pyrolytic recovery, the filters were first sonicated in water for 3 min to remove the precipitated salts, and then they were subjected to a slower inductive heating ramp up to 700 °C for 3 min. This process enabled the full recovery of the filters after nebulizing 3 mL of cell medium supplemented with serum and liposomes for 10 consecutive cycles, without losing any interfacial evaporation efficiencies. As shown by the TOC measurements of the collected liquid in the condenser chamber (Figure 8B), an average TOC removal of 99.89% was achieved. The SEM images of the filter surface after 10 pyrolytic cleaning processes confirmed that the surface was clean, and its nanostructuration slightly increased (Figure 8C). Such enhanced roughness resulted in an even higher hydrophilicity, as shown by a faster droplet absorption (Figure 8D). In addition, the induction heating curves exhibited a slight increase in the efficiency after the pyrolysis (Figure 8E).

The interfacial evaporation process in the SFSSM filters is also compatible with higher airflow rates. To demonstrate this, nebulization experiments with cell medium supplemented with serum and liposomes (nebulization speed 0.25 mL min^−1^) were carried out at an airflow rate of 30 L min^−1^. In this case, the average power was raised to 38 W, to achieve a filter temperature between 60 °C and 65 °C. Under these conditions, the collected mass in the Petri dish was zero. Therefore, the full evaporation of the aerosol microdroplets was achieved, despite the two‐fold shorter air residence time inside the filter network.

To achieve higher airflow speeds, as those needed in HVAC systems, SFSSM filters with larger diameter would be needed. The scalability of the annealing process was demonstrated in 6 cm diameter filters, obtaining similar ferromagnetic transformation and superhydrophilicity (Figure 8F,G). For integration into HVAC systems, a coil design shift from cylindrical to pancake‐shaped, akin to those in induction cooktops, would be necessary to ensure homogeneous inductive heating. Nonetheless, the 20 mm diameter filters and driving electronics used in this study would be already suitable for portable and clinical respirators.

With the size of the SFSSM filters (20 mm diameter, 3 mm thickness) used in this work, it was possible to nebulize up to 6 mL of a solution containing cell medium, serum, and liposomes, before blocking the filter. Considering that the average number of viral aerosol particles is *ca*. 5·10^5^ m^−3^ in close spaces,^[^ ^29^ ^]^ and assuming an average droplet diameter of 2 µm, the equivalent total volume of aerosol liquid per cubic meter would be 2 µL. Therefore, the 6 mL nebulized volume required to block the filter would correspond to ca. 3000 m^3^ of filtered air. At a flow rate of 15 L min^−1^ (i.e., 22 m^3^ per day), this air volume would represent ca. 130 days of continuous filtering before filter blocking. Considering the capacity to regenerate the filter surface by pyrolytic cleaning at least 10 times, the SFSSM filters could provide air disinfection during several years.

With a cost of ≈€1.25 per unit in large volumes, these filters offer significantly extended service life and recyclability compared to HEPA filters, leading to a drastic reduction in waste disposal. The power consumption (ca. 30W) is in the same order of other active air disinfection technology, such as UV and ionizing systems. Moreover, the power electronics would be compatible with the ones of induction cook‐tops and, therefore, its volume cost can be very low (i.e., less than 100€, depending on the final power). However, at this stage, it is complex to compare the final real cost and consumption of our device with other existing technologies, as further simple optimizations can be achieved. For example, the operating temperature and, consequently, the power consumption of our technology, could be further reduced by increasing the filters surface area, which now is moderate (ca. 0.5 m^2^ g^−1^), thereby enabling an even more efficient interfacial evaporation at lower temperatures. It is also worth highlighting that the validation experiments preformed in this work with nebulization rates of 0.25 mL min^−1^ and airflows of 15 L min^−1^ are rather extreme, with aerosol microdroplet concentrations much higher to those typically found in closed spaces (5·10^5^ m^−3^). Actually, reduction of the nebulization rate of samples composed of cell medium with serum and liposomes to 0.05 mL min^−1^ enabled complete liquid evaporation at filter temperatures between 45 °C and 50 °C, with a TOC elimination of 99.9% (Figure S2, Supporting Information), and average power consumption of just 15 W.

On the other hand, the moderate voltage of the electronic driving circuit, the low temperature of the filters during operation and the lack of generated radicals, ensure higher safety with respect to high temperature, UV or ionizing systems. Regarding the electromagnetic safety of the induction electronics, the supply voltage (< 30 V_DC_) is well below the 60 V_DC_ threshold, generally considered as safety extra‐low voltage according to IEC standards, meaning that there is no risk of electric shock to humans. The electric current levels involved are moderate and confined to the coil circuit. The electromagnetic exposure is also low, as the coil confines the magnetic field to a very localized region, with rapid spatial decay. At the operation frequency of 92 kHz, the penetration depth in biological tissue is high, but the induced currents are negligible at these field strengths and distances, well below limits defined by the International Commission on Non‐Ionizing Radiation Protection (ICNIRP) and IEEE guidelines for human exposure. Due to the small coil size and limited power, the radiated electromagnetic interference (EMI) is very low. No interference with common electronic devices (computers, sensors) was observed during operation. Shielding and grounding of the setup could mitigate improbable couplings. Therefore, considering the low voltage, limited current, small coil geometry and operating frequency, the inductive heating system does not pose safety risks to humans nor significant interference risks to nearby electronic equipment under normal laboratory operation.

## 3 Conclusion

In conclusion, we have successfully developed a novel air disinfection technology that leverages low‐temperature interfacial liquid evaporation. The system is built on cost‐effective and robust stainless‐steel filters, which are annealed at 900 °C for 1 h under atmospheric conditions. The annealing process transforms the austenitic stainless steel microporous filters into ferromagnetic superhydrophilic filters with enhanced induction heating efficiency. This combination of features enables complete interfacial evaporation of the aerosol microdroplets upon contact with the filter surface, leading to the precipitation of all organic and inorganic content at a low operating temperature range of 60 °C to 80 °C. The system operates with remarkable energy efficiency, consuming an average of only 30 W, and produces only sterilized air and steam at the output.

The working conditions to achieve the low temperature interfacial water evaporation and precipitation were first validated by the nearly complete removal of the inorganic and organic content of nebulized samples containing cell medium supplemented with serum and liposomes, organic pollutants (tetracycline) or polymer microparticles mimicking microplastics.

The air sterilization efficiency was demonstrated by nebulizing high concentrations of three distinct viruses: i) SARS‐CoV‐2 pseudovirus, ii) SARS‐CoV‐2 XBB.1.5 Omicron, and iii) RSV. Our results consistently showed viral eliminations of 99.6% or higher, with the majority of viral loads falling below the detection limit of the ELISA assays used for quantification. Given the 10 µm average pore size of the dense 3D filters, we anticipate the system will be even more effective against larger pathogens such as bacteria and fungi, where mechanical trapping will provide a significant additional layer of defense.

A key advantage of this technology is its reusability. A pyrolytic self‐cleaning process fully restores the filter's surface for at least ten cycles, a process that can even enhance its wettability and induction heating efficiency. This feature not only ensures safe reuse but also drastically reduces waste. The annealing process can be easily scaled up to larger diameter filters adapted for HVAC applications. However, the design of the induction coil and electronics would need to be re‐optimized for homogeneous filter heating. Further reductions in working temperature and power consumption could be achieved by using filters with a higher surface area to maximize the interfacial evaporation processes.

The convergence of low cost, robustness, scalability, reusability, minimal power consumption, and small footprint, with its ability to operate effectively without generating harmful radicals or requiring dangerous high voltages or temperatures, endows this air disinfection technology with substantial industrial potential.

## 4 Experimental Section

### Filter Preparation

The microporous sintered austenitic stainless steel (AISI 316L) filters (Amespore; 20.0 mm diameter and 3.0 mm thickness) with average pore size of 10 µm were purchased from Ames Group Sintering.

Two types of furnaces were used for the annealing and pyrolytic processes: a Lindberg Blue Mini‐Mite TF55030C‐1 tube furnace and an Equilab EQH‐3.0 induction furnace. The filters annealing was carried out inside a quartz tube under atmospheric conditions, at 950 °C for 60 min. The heating ramp was adjusted to 60 °C min^−1^ until the working temperature (950 °C) was reached. After the annealing time, the filters were slowly cooled down to room temperature. Alternatively, the heat treatment was carried out in an induction furnace, which makes the annealing process more energy efficient and economical compared to the tube furnace. In this case, the 950 °C annealing temperature was reached in ca. 40 s.

### Filter Characterization

The morphology and elemental composition of the filters were analyzed using a field emission scanning electron microscope (FE‐SEM FEI Quanta 650FEG, equipped with EDX detector). The chemical composition and oxidation state of the surface was analyzed by X‐ray photoelectron spectroscopy (XPS) (SPECS PHOIBOS 150 with a monochromatic X‐ray source Al K line = 1486.74 eV, 350 W). The spectral analysis was carried out with Casa XPS software and the spectra were calibrated to the binding energy (BE) of the C 1s peak (BE = 284.8 eV). The surface hydrophilicity was studied by contact angle (DSA25S Drop Shape Analyzer). The surface area and pore size of the substrates were determined by the Brunauer‐Emmett‐Teller (BET) method on a Micrometrics Tristar‐II.

The magnetic properties of the filters were measured using a Vibrating Sample Magnetometer with a maximum field of 15 kOe. Their dynamic magnetization under an alternating magnetic field was measured by an AC Magnetometer coupled to an optic fiber temperature sensor (Neoptix).^[^ ^30^ ^]^

The induction heating system was based on a custom‐made ZVS (Zero Voltage Switching) circuit with an LC resonant oscillator, optimized to minimize the switching losses and maximize the efficiency. The circuit operated at a frequency of 92.5 kHz. The electronics drive a hollow coil with 6 turns, responsible for generating the alternating magnetic field, yielding a maximum field of 40 mT, with a AC power consumption of 150 W. The filter temperature was monitored by either a thermal camera (FLIR A35 Thermal Imaging Camera, 30Hz, 69° FOV) or an infrared thermometer (CJMCU‐MLX90614 Infrared Thermometer), taking into account the infrared emissivity of the annealed filters, which was 0.81.

The samples nebulizations were carried out with a piezoelectric nebulizer (5V DIY‐1 V3.0 2023/1/30, Ultrasonic Piezoelectric Chip), connected to a syringe pump (LA‐110, RS232) to control the nebulization rate. The airflow was controlled with a diaphragm pump (GM‐1.00A) and a manual flow meter (LZQ‐5 Flowmeter 2.5‐25 LPM).

The mass of the collected liquids in the Petri dish and condenser chamber was measured with a precision balance (Ohaus Balance Discovery DV215CD).

The nebulized test liquids were composed of Roswell Park Memorial Institute (RPMI) 1640 Medium supplemented with 10% fetal bovine serum (FBS) media purchased from Thermo Fisher. The 100 nm diameter liposomes were obtained by thin‐film hydration method.^[^ ^31^ ^]^ Briefly, 1,2‐dioleoyl‐sn‐glycero‐3‐phosphocholine (DOPC, 8 µmol) and cholesterol (2 µmol) were mixed in 2 mL 2:1 chloroform:methanol. The organic solvent was evaporated under reduced pressure in a rotatory evaporator (Heidoph, Germany) leading to the formation of a thin film in the round flask. Then, the film was hydrated with 2 mL PBS pH 7.4 followed by three cycles of vortex (2 min) and sonication (1 min). The resulting white suspension was extruded 21 times through a 100 nm polycarbonate membrane (Avanti Polar Lipids, LLC) to get uniform size unilamellar liposomes.

The test samples with contaminants were composed of deionized water with either tetracycline (Sigma) at 16 ppm concentration at pH 8, or carboxylate‐polystyrene nanoparticles (Invitrogen) with a diameter of 100 nm, at a concentration of 100 µg mL^−1^. The absorbance spectra were measured by UV–vis spectroscopy (Carey 4000).

The total organic content of the samples was determined by TOC‐VCSH Shimadzu.

The conductivity of the collected samples was measured by conductivity meter (SensION+ EC7).

The pyrolysis cleaning process was carried out at 900 °C for 1 min in the Lindberg Blue Mini‐Mite TF55030C‐1 tube, or the Equilab EQH‐3.0 induction furnace at different temperatures, under atmospheric conditions. To minimize the risk of mechanical deformation of the filters, a milder cleaning process was optimized by first sonicating the filters in water for 3 min, followed by an induction heating ramp up to 700 °C for 3 min.

### Pseudovirus and Virus Production and Detection

Ethics statement: The biologic biosafety committee of the Research Institute Germans Trias i Pujol approved the execution of SARS‐CoV‐2 experiments at the BSL3 laboratory and the execution of RSV experiments at the BSL2 of the Centre for Comparative Medicine and Bioimage (CMCiB) with reference approvals CSB‐25‐008.

### Pseudovirus and Virus Production and Detection—Cell Model

Vero E6 cells (ATCC CRL‐1586), HEK‐293T (ATCC repository) and HEK‐293T overexpressing the human ACE2 (Integral Molecular Company) were cultured in Dulbecco's modified Eagle medium (DMEM; Gibco) supplemented with 10% fetal bovine serum (FBS), 100 U mL^−1^ penicillin, 100 µg mL^−1^ streptomycin (all from Invitrogen). HEK‐293T overexpressing the hACE2 was also complemented with 1 µg mL^−1^ of puromycin (Invitrogen).

### Pseudovirus and Virus Production and Detection—SARS‐CoV‐2 Pseudovirus Production

Single‐cycle infectious pseudoviruses were generated by co‐transfection (X‐tremeGENE HP Transfection Reagent; Merck) of HEK‐293T cells for 48h with two plasmids: SARS‐CoV‐2 SctΔ19 spike protein (Geneart) and HIV‐1 delta envelope reporter virus containing luciferase (pNL4‐3 Luc.R‐E; from NIH AIDS repository). Pseudovirus was titrated on HEK‐293T hACE2 cells with Bright Glo Luciferase system (Promega) using an Ensight Multimode Plate Reader (Perkin Elmer). All stocks were kept at −80 °C until use.^[^ ^32^ ^]^

### Pseudovirus and Virus Production and Detection—SARS‐CoV‐2 Virus Production

SARS‐CoV‐2 Omicron XBB.1.5 variant was isolated from a nasopharyngeal sample of a confirmed COVID‐19 patient. The virus was grown in Vero E6 cells (ATTC collection) at the BSL3 (NCB3) of CMCIB and sequenced (GISAID; EPI_ISL_17 308 812). SARS‐CoV‐2 virus was titrated in Vero E6 cells (Reed‐Muench method). All stocks were kept at −80 °C until use.^[^ ^32^ ^]^

### Pseudovirus and Virus Production and Detection—RSV Production

Viral stock was generated from an RSV strain A2 modified to express enhanced GFP (GFP5) from a gene cassette placed between the P and M genes (RSV‐GFP5 #R125; ViraTree). The virus was grown in Vero E6 cells (ATTC collection) at the BSL3 (NCB3) of CMCIB and titrated in Vero E6 cells (Reed‐Muench method). All stocks were kept at −80 °C until use.^[^ ^33^ ^]^

ELISA was used for detecting SARS‐CoV‐2 pseudovirus that were constructed by pseudotyping HIV‐1 particles with a SARS‐CoV‐2 spike. For this, the p24 gag ELISA INNOTEST HIV antigen mAb (FUJIREBIO, ref 80 563) was analyzed. For SARS‐CoV‐2 nucleocapsid (NC) the Human SARS‐CoV‐2 N ELISA Kit (Thermo Fisher Scientific, ref EH490RB) was employed, and, for RSV, the GFP ELISA Kit (ABCAM, ref ab171581). Initial viral input was 2897 ng mL^−1^ of p24 gag for SARS‐CoV‐2 pseudoviruses, 13.7 ng mL^−1^ of NC for SARS‐CoV‐2, and 4.56 ng mL^−1^ of GFP for RSV. Cell culture media was used as a negative control. The viral output for every non‐nebulized and nebulized condition was measured following the manufacturer's instructions. Mean viral protein ± SD (ng mL^−1^) detected on viral stocks with values above the background (negative control) were compared to nebulized samples to detect viral reduction upon filtering. Viral datasets were normalized by transforming each corresponding positive control to 100%, and subtracted 100 from each obtained value determine the removal efficiency. Statistical significance was measured with a one‐sample t‐test with Graph Prism v10.4.2.

## Conflict of Interest

The authors declare no conflict of interest.

## Supporting information



Supporting information

## Data Availability

The data that support the findings of this study are available on request from the corresponding author. The data are not publicly available due to privacy or ethical restrictions.

## References

[1] F. Liu , Q. Ma , M. M. Marjub , A. K. Suthammanont , S. Sun , H. Yao , Y. Tao , W. Zhang , ACS EST Eng. 2023, 3, 602.

[2] Y. Li , L. Wei , J. Lin , Z. Xie , L. Lu , X. Pan , J. Xu , R. Cai , J. Appl. Microbiol. 2024, 135, lxae078. 38520159 10.1093/jambio/lxae078

[3] G. Berry , I. Beckman , H. Cho , J. Aerosol. Sci. 2023, 167, 106078.

[4] S. Sankurantripati , F. Duchaine , Fluids 2024, 9, 281.

[5] M. Chen , Q. Hu , X. Wang , W. Zhang , Sep. Purif. Technol. 2024, 330, 125404.

[6] J. Malloy , A. Quintana , C. J. Jensen , K. Liu , Nano Lett. 2021, 21, 2968. 33759526 10.1021/acs.nanolett.1c00050

[7] Y. Zhang , Y. Zhao , J. Li , Y. Huang , Geomat. Nat. Hazards Risk 2024, 15,2322482.

[8] J. Akhtar , A. L. Garcia , L. Saenz , G. Garcia , S. Kuravi , F. Shu , K. Kota , A. Garcia , Indoor Air 2025, 2025, 1785997.

[9] H. Luo , L. Zhong , Build. Environ. 2021, 197, 107852. 33846664 10.1016/j.buildenv.2021.107852 PMC8021448

[10] Y. Kebbi , A. I. Muhammad , A. S. Sant'Ana , L. do Prado‐Silva , D. Liu , T. Ding , Compr. Rev. Food Sci. Food Saf. 2020, 19, 3501. 33337035 10.1111/1541-4337.12645

[11] K. Kompatscher , J. M. B. M. van der Vossen , S. P. M. van Heumen , A. A. L. Traversari , J. Hosp. Infect. 2023, 142, 39. 37797657 10.1016/j.jhin.2023.08.026

[12] S. A. Landry , M. Jamriska , V. J. Menon , L. Y. Y. Lee , I. Magnin‐Bougma , D. Subedi , J. J. Barr , J. Monty , K. Kevin , A. Gunatilaka , M. Delaire , G. B. Marks , A. J. Stewardson , L. Morawska , B. A. Edwards , S. S. Majumdar , K. Subbarao , S. A. Joosten , J. Hazard. Mater. 2025, 487, 137211. 39847930 10.1016/j.jhazmat.2025.137211

[13] M. G. Stanford , J. T. Li , Y. Chen , E. A. McHugh , A. Liopo , H. Xiao , J. M. Tour , ACS Nano 2019, 13, 11912. 31560513 10.1021/acsnano.9b05983

[14] J. T. Kim , J. Kwon , H. Lee , C. Kim , G. G. Yang , G. San Lee , C. W. Lee , J. G. Kim , S. Cha , H.‐T. Jung , S. Padmajan Sasikala , S. O. Kim , ACS Nano 2024, 18, 6387. 38364103 10.1021/acsnano.3c11284

[15] F. Liu , Q. Ma , J. Zhang , J. Wang , D. Govindan , M. Zhao , C. Gao , Y. Li , W. Zhang , ACS Appl. Mater. Interfaces 2025, 17, 27167. 40273420 10.1021/acsami.5c02969

[16] R. Dvorsky , J. Bednář , Z. Vilamová , Z. Šimonová , L. Svoboda , Sep. Purif. Technol. 2025, 360, 131002.

[17] J. Fu , T. Liu , S. S. Binte Touhid , F. Fu , X. Liu , ACS Nano 2023, 17, 1739. 36683285 10.1021/acsnano.2c08894

[18] S. W. L. Mah , D. P. Linklater , V. Tzanov , P. H. Le , C. Dekiwadia , E. Mayes , R. Simons , D. J. Eyckens , G. Moad , S. Saita , S. Joudkazis , D. A. Jans , V. A. Baulin , N. A. Borg , E. P. Ivanova , ACS Nano 2024, 18, 1404. 38127731 10.1021/acsnano.3c07099 PMC10902884

[19] M. S. Vlaskin , Appl. Therm. Eng. 2022, 202, 117855. 34867067 10.1016/j.applthermaleng.2021.117855 PMC8628600

[20] X. Zeng , C. Li , Z. Li , Z. Tao , M. Li , J. Environ. Sci. 2025, 154, 314. 10.1016/j.jes.2024.09.026 40049877

[21] L. Issman , B. Graves , J. Terrones , M. Hosmillo , R. Qiao , M. Glerum , S. Yeshurun , M. Pick , I. Goodfellow , J. Elliott , A. Boies , Carbon 2021, 183, 232.

[22] S. Lee , J. S. Nam , J. Han , Q. Zhang , E. I. Kauppinen , I. Jeon , ACS Appl. Nano Mater. 2021, 4, 8135. 37556284 10.1021/acsanm.1c01386

[23] S. Han , J. Kim , Y. Lee , J. Bang , C. G. Kim , J. Choi , J. Min , I. Ha , Y. Yoon , C.‐H. Yun , M. Cruz , B. J. Wiley , S. H. Ko , Nano Lett. 2022, 22, 524. 34665632 10.1021/acs.nanolett.1c02737

[24] X. Ji , J. Huang , L. Teng , S. Li , X. Li , W. Cai , Z. Chen , Y. Lai , Green Energy Environ. 2023, 8, 673.

[25] L. Yu , G. K. Peel , F. H. Cheema , W. S. Lawrence , N. Bukreyeva , C. W. Jinks , J. E. Peel , J. W. Peterson , S. Paessler , M. Hourani , Z. Ren , Mater. Today Phys. 2020, 15, 100249. 34173438 10.1016/j.mtphys.2020.100249 PMC7340062

[26] M. J. Esplandiu , N. Bastus , J. Fraxedas , I. Ihmaz , V. Puntes , J. Radjenovic , B. Sepúlveda , A. Serrá , S. Suárez‐García , G. Franzese , Encycl. Solid‐Liquid Interfaces. 2023, 3, 465.

[27] J. Nogués , B. Sepúlveda , M. J. Esplandiu , J. L. Tajada , WO/2024/003107, 2024.

[28] J. Nogués , B. Sepúlveda , M. J. Esplandiu , A. Fons , A. Lafuente , WO/2025/017138, 2025.

[29] A. J. Prussin , E. B. Garcia , L. C. Marr , Environ. Sci. Technol. Lett. 2015, 2, 84. 26225354 10.1021/acs.estlett.5b00050 PMC4515362

[30] E. Garaio , J. M. Collantes , F. Plazaola , J. A. Garcia , I. Castellanos‐Rubio , Meas. Sci. Technol. 2014, 25, 115702.

[31] D. Lombardo , M. A. Kiselev , Pharmaceutics 2022, 14, 543. 35335920 10.3390/pharmaceutics14030543 PMC8955843

[32] J. Rodon , J. Muñoz‐Basagoiti , D. Perez‐Zsolt , M. Noguera‐Julian , R. Paredes , L. Mateu , C. Quiñones , C. Perez , I. Erkizia , I. Blanco , A. Valencia , V. Guallar , J. Carrillo , J. Blanco , J. Segalés , B. Clotet , J. Vergara‐Alert , N. Izquierdo‐Useros , Front. Pharmacol. 2021, 12, 646676. 33841165 10.3389/fphar.2021.646676 PMC8033486

[33] E. Molina Molina , J. J. Bech‐Serra , E. Franco‐Trepat , I. Jarne , D. Perez‐Zsolt , R. Badia , E. Riveira‐Muñoz , E. Garcia‐Vidal , L. Revilla , S. Franco , F. Tarrés‐Freixas , N. Roca , G. Ceada , K. Kochanowski , D. Raïch‐Regué , I. Erkizia , R. Boreika , A. E. Bordoy , L. Soler , S. Guil , J. Carrillo , J. Blanco , M. Á. Martínez , R. Paredes , A. Losada , P. Aviles , C. Cuevas , J. Vergara‐Alert , J. Segalés , B. Clotet , Nat. Commun. 2025, 16, 1087. 39920115 10.1038/s41467-025-56151-y PMC11805953

