# Supplementary material for: Highly Efficient Air Sterilization via Low‐Temperature Interfacial Evaporation in Inductively Heated Superhydrophilic Ferromagnetic Filters
Source: Adv Sci (Weinh). 2025 Sep 28;12(44):e09118. doi: 10.1002/advs.202509118 (PMC12667521; doi:10.1002/advs.202509118)
Supplement: Supplementary file 1 — Supporting information [file ADVS-12-e09118-s001.docx]

Supporting Information

Highly Efficient Air Sterilization Via Low-Temperature Interfacial Evaporation In Inductively Heated Superhydrophilic Ferromagnetic Filters

*A. Fons,^1,&^ C. Vaca,^1,&^ E. Franco-Trepat,^2,&^ A. Lafuente,^3^ J. L. Tajada,^3^ A. Serrà,^4^ J. D. Pedroza,^5^ S. Franco,^2^ R. Boreika,^2^ I. Erkizia,^2^ N. Izquierdo-Useros,^2,6^ A. López-Ortega,^7,8^ E. Garaio,^7,8^ M. J. Esplandiu,^3^* *J. Nogues,^3,9^ and B. Sepúlveda*^1^*

**Content**

Figure S1: Inductive heating at higher flow rates.

Table S1: ELISA quantification of p24 gag protein before and after lysis buffer on the SARS-CoV-2 pseudovirus samples

Table S2: ELISA quantification of p24 gag proteins from SARS-CoV-2 pseudovirus samples after lysis buffer treatment.

Table S3: ELISA quantification of nucleocapsid proteins from SARS-CoV-2 Omicron virus samples after lysis buffer treatment.

Table S4: ELISA quantification of GFP proteins from RSV samples after lysis buffer treatment.


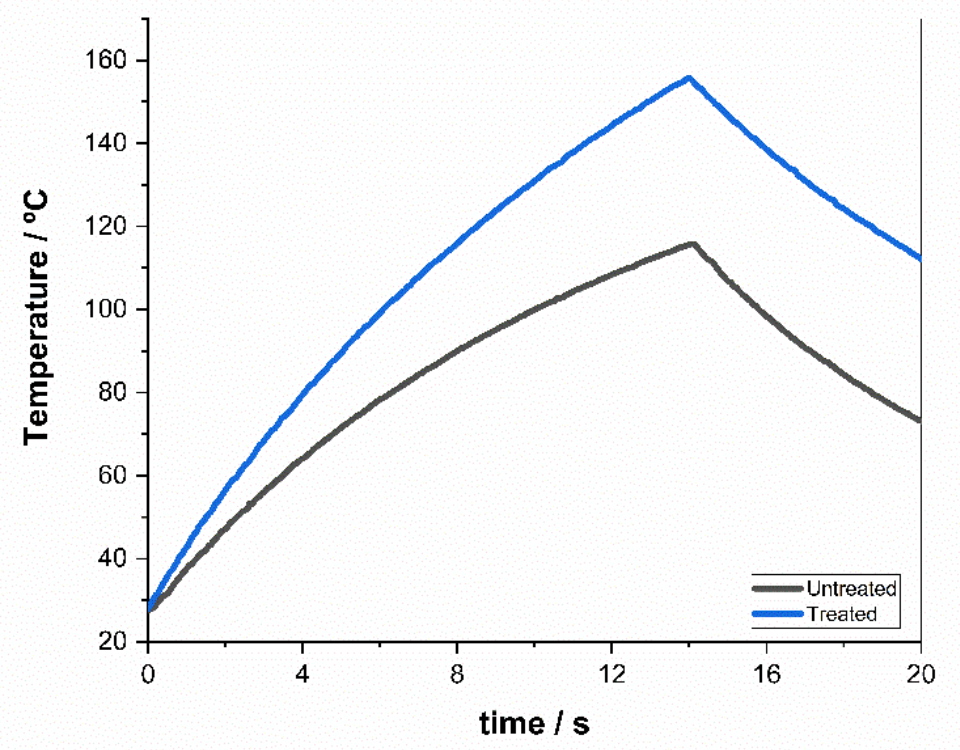


**Figure S1. Inductive heating at higher flow rates.** Comparison of inductive heating under airflow rate of 20 L/min for untreated and annealed filters at 950°C for 1 h.

**Table S1. ELISA quantification of p24 gag protein before and after lysis buffer on the SARS-CoV-2 pseudovirus samples**: i) positive control and ii) sample collected the Petri dish after nebulization of the pseudovirus at 0.25 mL/min when the filter is kept at room temperature.

|  | **p24 gag concentration (ng/mL) before lysis buffer** | **p24 gag concentration (ng/mL) after lysis buffer** | **Ratio before/after lysis buffer** |
| --- | --- | --- | --- |
| **Positive control** | 132.508 | 848.71 | 0.15 |
| **Nebulization at RT** | 216.44 | 1234.35 | 0.17 |

**Table S2. ELISA quantification of p24 gag proteins from SARS-CoV-2 pseudovirus samples after lysis buffer treatment.** We compared positive and negative controls with the samples collected in the condenser chamber after nebulization of the pseudovirus at 0.25 mL/min while keeping the filter at a temperature between 60 and 80°C.

|  | **p24 gag concentration (ng/mL) after lysis buffer** | | |
| --- | --- | --- | --- |
|  | **Replica 1** | **Replica 2** | **Average** |
| **Negative control** | 0.02 | 0.02 | 0.02 |
| **Positive control** | 3168.00 | 2626.46 | 2897.23 |
| **Nebulization 1 at 70-80°C** | 0.37 | 0.37 | 0.13 |
| **Nebulization 2 at 70-80°C** | 0.04 | 0.04 |  |
| **Nebulization 3 at 70-80°C** | 0.10 | 0.09 |  |
| **Nebulization 4 at 70-80°C** | 0.00 | 0.00 |  |

**Table S3. ELISA quantification of nucleocapsid proteins from SARS-CoV-2 Omicron virus samples after lysis buffer treatment**. We compared positive and negative controls with samples collected in the condenser chamber after nebulization of the virus at 0.25 mL/min while keeping the filter at a temperature between 60 and 80°C.

|  | **nucleocapsid concentration (ng/mL) after lysis buffer** | | |
| --- | --- | --- | --- |
|  | **Replica 1** | **Replica 2** | **Average** |
| **Negative control** | 0.00 | 0.00 | 0.00 |
| **Positive control** | 13.71 | 13.05 | 13.38 |
| **Nebulization 1** | 0.00 | 0.08 | 0.03 |
| **Nebulization 2** | 0.00 | 0.00 |  |
| **Nebulization 3** | 0.00 | 0.18 |  |
| **Nebulization 4** | 0.00 | 0.00 |  |

**Table S4. ELISA quantification of GFP protein of the RSV samples after lysis buffer treatment**. We compared positive and negative controls with samples collected in the condenser chamber after nebulization of the virus at 0.25 mL/min while keeping the filter at a temperature between 60 and 80°C.

|  | **GFP concentration (ng/mL) after lysis buffer** | | |
| --- | --- | --- | --- |
|  | **Replica 1** | **Replica 2** | **Average** |
| **Negative control 1** | 0,000 | 0,000 | 0,001 |
| **Negative control 2** | 0,000 | 0,003 |  |
| **Positive control 1** | 5,535 | 3,768 | 4,561 |
| **Positive control 2** | 5,371 | 3,571 |  |
| **Nebulization 1** | 0,000 | 0,000 | 0,21 |
| **Nebulization 2** | 0,004 | 0,000 |  |
| **Nebulization 3** | 0,102 | 0,000 |  |
| **Nebulization 4** | 0,058 | 0,000 |  |

**

**

**Figure S2. TOC value of the condensed liquid at a nebulization rate of 0.05 mL/min.** The airflow speed was 15 L/min and power consumption 15 W, giving a filter temperature between 45 ºC and 50 ºC.
